# Etiology of Two Outbreaks of Disease among Hogs1The writer is much indebted to Dr. Austin Peters for advice and assistance, to the Massachusetts Society for the Promotion of Agriculture for animals and use of farm, and to the Harvard Medical School for laboratory facilities.

**Published:** 1890-12

**Authors:** John Amory Jeffries


THE JOURNAL
OF
COMPARATIVE MEDICINE AND
VETERINARY ARCHIVES.

VOL. XI. DECEMBER, 1890. No. 12.

### ETIOLOGY OF TWO OUTBREAKS OF DISEASE AMONG HOGS.[1]

By JOHN AMORY JEFFRIES, M.D.

DURING the last of February, 1889, my attention was called by Dr. Peters, D.V.S., to an epizoötic among hogs on a farm near Boston. The disease belonged to the group commonly called hog cholera, red soldier, swine plague and other names. The leading symptoms were a red or purple skin, fever, diarrhœa, cough and short, rapid respiration. Nearly or quite all the pigs were affected, and the mortality rose to about 50 per cent. Pigs not dying made a slow recovery.

Later in the year a flock of sheep on the same farm were affected with much the same set of symptoms, and the greater part died.

Of such outbreaks among hogs Dr. Peters has seen six, while I have found three or four more. In nearly every case the hogs have been fed on city swill. This, the farmers believe, will kill hogs, if not boiled before feeding.

One dead, full-grown hog was sent to me for bacteriological examination. Unfortunately, decomposition was well under way before it came to hand. Autopsy made by Dr. Peters and myself showed skin red and blue in patches and studded with ecchymoses, especially on the ventral surface. Lungs dark red to

[1] The writer is much indebted to Dr. Austin Peters for advice and assistance, to the Massachusetts Society for the Promotion of Agriculture for animals and use of farm, and to the Harvard Medical School for laboratory facilities.

purple, resistant, but not so solid as in lobar pneumonia; cut surface moist, bloody fluid, with few bubbles oozing out on pressure; bloody pleural and pericardial effusions; pleuræ, pericardium and peritoneum cloudy and studded with small hemorrhages; spleen enlarged, emphysematous, putrid; liver congested; kidneys with hemorrhagic blebs up to the size of small beechnuts scattered over the surface; intestines much decomposed, so that the presence of ulcerations cannot be affirmed.

Microscopic examination of lungs showed a moderate amount of catarrhal pneumonia. Sections of the abdominal organs stained very poorly, and showed little beside marked cell atrophy about the points of hemorrhage. In all the organs three kinds of bacteria were found—a large spore containing drumstick bacillus, a fair-sized, short bacillus and the Schutz-Loeffler swine-plague germ. The presence of all three forms was easily demonstrated by the cover-glass method. In sections, however, the short bacillus stained but poorly, and the swine-plague bacillus with great difficulty. Hæmatoxylin, Loeffler's solution, and safronin gave the best results. Careful examination showed that the drumstick bacillus and the short one bore no relation to the pathological changes. The first varied in abundance in proportion to the degree of putrefaction; the second was scattered in small clumps all through the organs. The Loeffler germ, however, occurred only in the vicinity of marked pathological changes, then in great numbers.

Gelatin plate cultures were made from subcutaneous tissue, blood and organs; also agar-agar test-tube cultures were put in the thermostat. The latter gave mixed cultures of the short bacillus and the Loeffler germ; the former developed two kinds of colonies, the first at the end of thirty-six hours, as pin-head, white colonies, few in number; the second (Loeffler) appeared as very numerous pin-point colonies on the fourth day. From the different colonies agar-agar cultures were made, and from these plates, to insure purity.

*Animal Inoculations.*—February 28th, 1889, inoculated guinea-pig with one of the agar cultures into the abdomen. On the morning of March 1st the pig was found dead.

*Autopsy.*—Extensive accumulation of clear fluid, coagulating on exposure to the air, in peritoneum. Peritoneum cloudy, studded with a few small hemorrhages; liver congested, soft, enlarged; spleen slightly enlarged; kidneys enlarged, soft, cloudy, streaked with small hemorrhages; lungs engorged, œdematous, with small spots of lobular pneumonia.

Cover-glass preparations and gelatine plate cultures showed the presence of both the short bacillus and Loeffler germ.

Another guinea-pig was inoculated with fluid from the abdominal cavity of the first. It died during the night, and presented the same autopsy. From this a third guinea-pig was inoculated, which also presented the same autopsy and bacteriological results. Finding that both the short bacillus and the Loeffler germ were going through the guinea-pigs, I fell back on the pure cultures from the original hog, and quickly found that the short bacillus had no effect on guinea pigs, white rats or pigeons, even when whole cultures were introduced into the abdominal cavity. On the other hand, one drop of a milky fluid of the Loeffler germ killed guinea-pigs within thirty-four hours, if introduced into the abdomen, and in from three to five days if under the skin.

The full summary of results of inoculation experiments will be given later on.

About this time two hogs were inoculated each with an agar-agar culture, one under the skin and one into the abdomen. These seemed a little dumpy for a day or two, but never showed any marked symptoms. At the same time a hog was inoculated with a very large culture of the short bacillus. It remained perfectly well.

Nothing more was done until February 3d, 1890, when two small, live, sick hogs were received from Holyoke, Mass. They were apparently suffering from the same disease as the first hog. They were killed and examined. The autopsies revealed in one a red skin, a number of small ulcers in the vicinity of the cæcum and enlarged mesenteric glands; in the other, intestine the same, marked lobular pneumonia of lower lobes and spleen four times enlarged. A series of gelatin plates and a series of agar-agar tube cultures remained sterile, except for a couple of contaminating colonies. On investigation, it was found that the gelatin was barely alkaline, did not take when inoculated with the Löffler germ from the first hog, but did when inoculated from a culture of swine plague from Dr. Billings. No cover-glass preparations were made.

From subsequent experience with cultures of Loeffler's germ and hog cholera I am quite confident that the former was present in the two hogs.

On March 17th, 1890, I received two more young hogs from the same farm as the first hog. One had died on the way; the other was still living.

Autopsy of the dead hog showed skin of ventral surface almost purple; inflammation of large and small intestines; in cæcum spots denuded of epithelium but no well-marked ulcers; mesentric glands enlarged. Pericarditis; extensive catarrhal pneumonia of both lungs.

The living hog was killed by a blow on the head and also autopsied. Skin slightly reddened; lymphatic glands of groins, axillæ and neck enlarged. Intestines slightly reddened and inflamed in spots, no true ulcerations; spleen enlarged, soft with small petichiæ; glands of mesentery enlarged. Left fibrinous, pleuritis and lobular pneumonia; right pleura and lung normal.

Nothing abnormal could be detected in the other organs of either hog.

Gelatin plates and agar-agar cultures developed, with the exception of two contaminating colonies, only the Loeffler germ.

A piece of the spleen and a lymphatic gland of pig 2 were mashed in a mortar with some bouillon and thirty minims injected into the abdominal cavity of a guinea-pig. It died in twenty-four hours.

*Autopsy.*—Peritoneum cloudy; increase of fluid with fibrin flocculi: veins of mesentery engorged; spleen not enlarged; kidneys swollen, hyperæmic; liver engorged; lungs, lower lobes engorged, dark red, partly contracting; two hemorrhagic imtacts in right lung. Lymphatics not enlarged; vessels of skin engorged.

Cover-glass preparations, plate cultures, and agar-agar cultures developed pure culture of the Loeffler germ in great numbers.

Inoculation experiments on young hogs were made by Dr. Peters as reported below. These were later killed and examined. Cover glass preparations from all four showed the germ in the spleen and glands of mediastinum. Cultures were only made from the first of the four pigs. From these, pure cultures of the Loeffler germ were got.

A calf placed in an adjacent pen to the experiment pigs, in two days became sick, and was killed while in a moribund state, by Dr. Peters. Cover-glass preparations showed the presence of the Loeffler germ. Cultures from abdominal organs gave only Loeffler's ; the plates from the lung showed the presence of a large micrococcus (inert) and a few colonies of S. P. aureus.

NOTES ON SWINE-DISEASE EXPERIMENTS AT THE FARM, WALK-HILL STREET, MATTAPAN, MADE BY DR. PETERS.

PIGS.—Four little pigs, six or eight weeks old, bought for the purpose in the neighborhood, appear bright and healthy. Pigs Nos. 1 and 2 treated the same except the dates of killing them. March 26th.—Four guinea-pigs inoculated by Dr. Jeffries, and taken out to the farm. March 27th.—The guinea-pigs died ; were skinned and the carcasses chopped up and mixed in the food of these pigs (Nos. 1 and 2). By April 2d, Nos. 1 and 2 were a little dull for a day, but afterwards were bright and lively again. April 17th.—Carried four flasks containing bouillon, in which cultures had been growing a few days in constant temperature apparatus, out to the farm, a flask of the culture to be emptied into the pigs' milk at night and fed to them the next morning, the feeding commencing April 18th. April 18th.—Took four more inoculated flasks of bouillon to the farm ; these eight flasks were emptied in the pigs' milk at night and fed the next morning, the last of these being fed April 25th. April 26th.—Took out two more flasks of bouillon which had been in the thermostat up to this time ; instead of pouring the contents into the milk the night before, the culture was put in the milk just before feeding in the morning. The culture when put in the milk over night caused it to become darkish colored, and often a black scum was found on the milk. The last two cultures were fed in the morning on April 27th and 28th. May 3d, Saturday.—The pigs Nos. 1 and 2 were noticed to have red spots on the ears, the epithelium peeling off the edges of the ears, and a few red patches were also on the backs and sides ; they do not eat well, are lying down nearly all the time, and if roused and made to move around the pen they cough a great deal.

May 6th.—Killed pig No. 1, Dr. Jeffries being present and making cultures from various organs. Before killing it the following symptoms were observed in addition to those of May 3d :

The breathing was much accelerated and accompanied by a husky sound, coughed frequently, and had diarrhœa, the feces being much softened, clay-colored, and very offensive to the smell. Pig No. 2 also had a diarrhœa on this date similar to pig No. 1.

*Autopsy on Pig No. 1.*—There were patches of lobular pneumonia in both lungs; catarrh of the smaller bronchi; both layers of the pericardium were thickened and opaque in spots. In the liver were a number of nodules, and its capsule was opaque in places; these nodules may or may not be tuberculous, to be determined by Dr. Jeffries. On opening the ileum and cæcum, the Peyer's patches appeared very prominent, but were not ulcerated. The spleen and kidneys were apparently normal.

PIG No. 2.—May 13.—Pig No. 2 has coughed less every day for several days. The diarrhœa continued (after May 6th) for four or five days, becoming if anything worse, and the odor of the feces being more offensive; but the last two or three days he has improved very rapidly and is now nearly well, except coughing occasionally. May 15th.—Pig No. 2 was killed. Autopsy revealed extensive catarrhal pneumonia of both lungs, enlarged mediastinal and mesenteric lymphatics; signs of pericarditis; spots in liver as in pig No. 1; spleen, kidneys and intestines apparently normal. For several days prior to being killed, pig No. 2 had been improving, eating much better and diarrhœa ceasing. Cover glass preparations were made from mediastinal lymphatic, blood from the heart, and the spleen.

PIG No 3.—March 26th.—I inoculated pig No. 3 with some of the original culture, mixing the culture from test tube of agar with water and injecting a syringeful into the abdomen, and another syringeful under the skin on abdomen; the syringe was a small one holding only about 2 cc., and did not work very well, and did not get much in. March 28th.—Pig has not eaten its breakfast, and the farmer says it does not feel well, but I cannot see that it appears sick except the loss of appetite. Pig seemed very well again until April 2d, when it was a little dull for a day, after which it improved. Pig 4, used so far as a control pig, was on April 2 bright and lively.

About April 14th all the pigs were removed from the calf pens where they were, to a pig house outside, because I thought they made two calves sick in adjoining pens. The pigs were separated in the pig house by a low board partition, Nos. 1 and 2 being in one half and Nos. 3 and 4 in the other. Pig No. 3 did very well until May 8th or 10th, when it again became a little dull and coughed a good deal; this was about five or six days after pigs 1 and 2 were taken sick as a result of the feeding experiments, and I think it was infected this time by being in a pen next them. No diarrhœa noticed.

May 15th.—Killed pigNo. 3. Autopsy revealed in the lungs (both) a few small spots of lobular pneumonia and also some patches of fine bronchitis, a few opaque spots in both layers of the pericardium, enlarged mediastinal and mesenteric lymphatics, and a few opaque spots in the capsule of the liver; the viscera appeared normal. Cover glass preparations made from mediastinal lymphatic glands.

Pig No. 4.—April 6th.—Inoculated this pig with culture from two agar tubes mixed with water; used a large hypodermic syringe holding about 5 cc., with a large needle, and every syringeful went into him. He received a syringeful into the abdomen, one subcutaneously over the abdomen, and one into the right lung, the needle of the syringe being inserted between the two ribs. April 8th.—Pig very ill, refusing food and staying curled up in the straw, refusing to move unless compelled to; breathing hurried and labored. For a day or two I thought he would die. April 10th.—Much better; bright and lively; eating well; breathes hard yet and coughs. From this time on pig 4 improved, excepting one day, April 16th, it was a little dull and did not eat, although he seemed pretty well until he was killed. He remained small and did not grow so fast as the rest.

May 15th.—Killed Pig No. 4.

*Autopsy.*—Found little spots of lobular pneumonia in the anterior lobes of both lungs, but no old signs of trouble at the point of inoculation of April 6th; there were traces of pericarditis as in the others, and an enlarged mediastinal lymphatic; otherwise the organs appeared healthy except that the Peyers patches around the ileo-cæcal valve were very prominent, although not ulcerated. Cover glass preparations made from mediastinal lymphatics, blood from heart and spleen.

Calves.—Two calves were bought and taken to farm April 7th for feeding with tuberculous milk. They were placed in pens adjoining the pigs, not thinking that the pigs were likely to infect the calves. The calves were a bull about two weeks old, and a heifer four or five days old.

April 9th, the heifer calf (calf 2), did not seem to feel well was dull and did not care for her milk. She grew steadily worse, and by April 12th had developed what appeared to be a case of double pneumonia, breathing rapid and labored; dulness on percussion on both sides. It continued to grow worse until April 16th, when, being in a moribund condition, it was killed.

Autopsy revealed pneumonia of the lower halves of both lungs, with spots of gray hepatization through the diseased portions; the pleura was thickened in spots, especially over part of the posterior lobe of the left lung, there was an enlarged lymphatic in the posterior mediastinum; the pericardium, both parietal and visceral were congested and the vessels injected. The intestines and mesentery were congested (but this may have been passive); the liver was engorged with blood, the capsule of the spleen was ingested, and the spleen seemed to contain more blood than usual, of a dark tarry consistency. Kidneys apparently normal.

Specimens were saved from the lungs and carried in to Dr. Jeffries. Mediastinal lymphatic (whole gland), liver, spleen, kidney and a mesenteric lymphatic gland.

The other calf was sick for several days; he did not seem to have any lung trouble, but was dull, had no appetite, his ears drooped and he had a little diarrhœa, feces being light-colored and offensive.

After changing the pigs to another place and taking away the heifer calf and disinfecting the pens, this calf began to improve, and it finally recovered, although it was a week or ten days after killing the calf and disinfecting the pens that this calf began to really feel well again.

Subsequently, many more guinea-pigs were inoculated with cultures derived from the first hog, the two last and the calf—in all between twenty-five and thirty. The results of the autopsies are summarized as follows:

Guinea-pigs, when inoculated with a few drops of a cloudy mixture into the abdomen, show signs of sickness in a few hours. They become more quiet, stop eating and even cuddle up in a corner. After a time the hair becomes erect, the skin red and the animal often shivers. Lastly, marked prostration occurs, the animal lies on its side panting heavily. I have never seen one die. Death occurs in from twelve to sixteen hours.

Autopsy shows a marked increase of fluid with fibrin flocculi in peritoneum. Peritoneum dull, sticky, not glistening. Spleen at times slightly enlarged, congested, but never much affected. Kidneys in about 50 per cent. are swollen, soft, dark in color, and on section give the appearance of cloudy swelling. Liver is more or less enlarged, soft, dark ; acini indistinct, rich in blood. In most cases the heart is full of dark blood, the muscles soft ; endocardium and pericardium cloudy, also much fluid in pericardium ; pluræ cloudy ; lungs, the most affected of any organs, show all stages, from that of marked engorgement to a state of catarrhal pneumonia, affecting the entirety of both lungs. I say catarrhal pneumonia, since the microscopic appearances resemble this condition in man and not that of lobar pneumonia. They contract but poorly, pit on pressure, crepitate but little ; pieces often sink at once in alcohol and vary from bright red to bluish in color. Cut surface shows the same range of color, is moist, and gives a fluid, bloody scraping, with few bubbles. Trachea not affected.

The state of the intestines is variable ; at times normal, at times slightly congested, more rarely decidedly so, but never approaching the condition induced by Brieger's bacillus. Though no diarrhœa was noted in the guinea-pigs, the rectum was often found at autopsy to be full of soft feces flowing from the body on the slightest disturbance.

A few of the guinea-pigs, notably those inoculated nearest the hogs, showed a more or less marked tendency to small hemorrhages. These have been found in lungs, liver, kidneys and peritoneum, also in subcutaneous tissue. Considerable œdema is liable to occur where the needle pierces the abdominal wall.

Cover glass preparations show the germ in great numbers in all the organs. Wherever leucocytes are present, these are very apt to contain the plant from one to vast numbers. The process is not that of phargocytosis, but just the reverse, the first stage being a leucocyte with one germ, the last stage a mass of germs with the form of a leucocyte. The nucleus is the last part destroyed. The process is most marked in the abdominal fluid. In the blood I have found but few germs and a reduction in the number of white corpuscles.

Sections show the germ scattered about and in clumps at points of hemorrhage or other marked changes, the impression given being that an infected leucocyte had stuck, been destroyed and then the adjacent cells attacked ; the nuclei resisting and staining well long after the cell body has vanished. Marked cell atrophy often occurs about the hemorrhages. In general, the cells of the abdominal organs are much more cloudy than is normal. The bacilli are also found singly. If the guinea-pig has been inoculated under the skin, the differences are local œdema; at times cheesy products at site of inoculation; adjacent lymphatics enlarged; little increase of fluid in peritoneum. The distribution of the germ is the same, but, of course, is less abundant in the peritoneum, while thronging at site of œdema. Death is slower, in from three to five days.

Two rabbits offered no differences. A white rat, with one drop subcutaneously, became very ill for three weeks, but recovered. Symptoms, those of pneumonia.

Two pigeons inoculated into the breast muscle became dumpy for a time. Later, inoculated into the abdomen, they died like the pigs, and, making allowances for difference in histology, the same pathological changes. The birds did not resemble those with chicken cholera; the eyes were not affected.

It will be seen that in the three hogs and calf three different germs were found—a drum-stick bacillus, a short bacillus and the germ which I have called Loeffler's. The drumstick form can be excluded at once on the following grounds: It is a well-known cause of putrefaction, and varies with the degree of decomposition; it occurred only in the pig dead for some time; it did not grow in guinea-pigs.

The short bacillus found in the same hog might be assumed to have some connection with the disease, but many weighty reasons preclude the idea. Though readily detected, it was found in but one hog. It had no effect on either guinea-pigs or hogs when inoculated in great quantities. Its distribution in sections evidently has no relation to the pathological changes in the organs of the hog.

There seems no doubt that its occurrence was an example of secondary infection, its existence in the animals being rendered possible by the effects of the Loeffler germ. Such secondary infection by germs, benign or not directly pathogenic, is well known and has been the cause of not a few errors. The form, growth, lack of motion and effect on guinea-pigs precluded all idea of the germ being that of hog cholera.

We have left, therefore, but one form, the Loeffler, as a possible cause of the disease. The evidence in its favor is very strong. It occurred in all three hogs; in two alone. It occurred in the infected calf. It killed guinea-pigs, rabbits and pigeons, with similar symptoms and lesions. It produced the same group of symptoms in healthy young hogs, and was alone found in their bodies after they had been killed.

As the whole question of disease among hogs and other domestic animals is involved in something like chaos, I give a detailed description of the plant as isolated by me and a comparison with all the material relating to the subject which I have been able to procure. No exhaustive study of the literature will be given, partly because all the originals are not accessible, but chiefly because the mass of the writings on the subject are of no value in determining the bacteria. This is due to the inherent difficulty of giving a description which will apply to but one kind of bacterium, careless work and the unwarranted assumption that all diseases of the hog are the same.

### DESCRIPTION OF BACTERIUM.

*Form.*—The plant belongs to that most confusing group of bipolar organisms. As seen in bouillon culture it appears as small micrococci about .4 $\mu$ in diameter, as two micrococci, with a space between—total length, 1 to 1.3 $\mu$—or as short bacilli, up to 1 $\mu$ long. When stained, cultures are very apt to give all the appearances of small micrococci, but a careful examination shows that many of them go in pairs, as if the ends of a bacillus 1 to 1.3 $\mu$ long. The nature of the nutrient does not seem to make any difference. Old cultures, however, are liable to show a certain number of bacilli and degenerative forms.

In animals, as shown by the study of cover-glass preparations, the form is about the same, appearing as small cocci and beautiful bipolar bacilli. The general impression is that of a vast number of micrococci. In the abdomens of rabbits threads are formed; none have been noted in guinea-pigs. No motion other than Brownian can be detected.

GELATINE PLATES.—The colonies appear some time between the third and seventh days, according to the nature of the gelatine and temperature of the room. They are white, pin-point spheres on and in the gelatine.

GELATINE TUBES.—Growth is slow, never extensive, white, As a rule, there is a limited growth on the surface of the gelatine, this is viscous and a long, tapering shaft along the needle tract. This, while very difficult to describe, is more or less characteristic, appearing as a light, gauzy lacework made up of many very minute spheres, in places blending.

The plant is very sensitive to the degree of reaction of the nutrient, often failing to grow, though red litmus is turned blue by the gelatine. Other bacteria, as the hog cholera (Salmon) or swine plague (Billings), do well in such gelatine. If a considerable amount of alkali be added to such gelatine, the plant will grow well. Fig. 8, Plate XI, Hog cholera, Bureau of Animal Industry, 1889, will do fairly well for a five days' culture, Fig. 4 being taken as hog cholera of the same age.

AGAR-AGAR.—Here the growth is more or less variable. At times it forms a moderately thick, white, semi-transparent growth along the line of inoculation, or spread over the surface, if moist. The growth is strongest on agar with glycerine. In my experience, however, the growth is in most cases very limited, thin, and transparent, at times almost invisible to direct view. Close study shows that the growth is composed, as it were, of very minute dewdrops. Cultures many generations old average less profuse than those direct from animals. On agar, as in gelatine, the plant is very sensitive to the degree of alkaline reaction. Bubbles have never been noted.

BOUILLON.—This shortly becomes cloudy, and remains so for a time, after which the growth sinks to the bottom as a fine cloud.

POTATO.—Personally, I have never been able to procure any growth on potato, in spite of many efforts. In nearly all cases cover-glass preparations were made, to preclude error. If my diagnosis is right, however, the plant has been known to grow on this medium.

WHITE OF EGG.—Growth thin, not spreading far from point of inoculation, viscous, with a trace of buff color.

For purposes of identification I have had, beside the literature—to my mind in this case a blind guide—the following material :

A series of sections from a hog dead of "schweinpest," prepared by me in Vienna in the Winter of 1886. No cultures.

These show a bipolar bacillus, well stained by the Gram method, occurring in clumps. A large series of sections from rabbits, and cover-glass preparations from cultures of Hueppes. Septicæmia hæmorrhagica or Wild seuche, prepared by Dr. Jackson, who assisted Hueppe in his work. A pure culture of hog cholera from Dr. Smith, also one of swine plague. Five pure cultures of swine plague from as many outbreaks, two unnamed cultures from one hog, and a culture of Schutz's germ from Dr. Billings.

Careful comparison of all the cultures has been made as grown on gelatine, agar-agar with 7 per cent. glycerine, potato, white of egg. Guinea-pigs have also been inoculated with the various germs, symptoms noted, careful autopsies made and the organs examined by section and cover-glass methods. In the case of each medium used all were of one lot, inoculated at one time and kept together,

In many trials the germ from all of my sources has never shown any difference; all are precisely alike, and all agree in every particular with cultures of swine plague (Smith).

Cultures of Schutz germ, *i. e.*, the Loeffler bacillus, agree precisely as regards form, lack of motion and general characters of growth, but are, on the average, a little more profuse in growth. The same is true of the unnamed culture from Dr. Billings, except that growth on gelatine may be a trifle stronger, and that on egg dryer. Cultures of hog cholera (Smith), swineplague (Billings), are precisely alike, and differ from my cultures in many particulars, though presenting a close superficial resemblance. In form they appear as short, relatively stout bacilli, often in two; at times very short, micrococcus like; at times fairly long. No bipolar structure is apparent in the living plant. When stained, this at times appears, but never, so far as noted, to such a degree as to resemble two micrococci. In cover-glass preparations the resemblance is closer, but the hog cholera germ averages larger—more of a bacillus. On gelatine the growth is very similar, but differs in being more profuse and quickly appearing. Colonies are distinct by thirty-six hours. Agar-agar cultures grow promptly, as a thick, white coat along needle track, and at least often form bubbles in the condensation water when glycerine is present. The plant is not sensitive to the degree of alkalinity of the nutrients used.

Bouillon cultures are more cloudy, sediment heavier and surface scum more pronounced.

On white of egg the hog cholera germ shows the same class of difference as on other media. It is much more profuse, softer and has a faint trace of yellow.

POTATO.—In my experience growth on potato is rather slow; visible about the fourth day; of a soft, creamy white.

All cultures grow well at the temperature of the room.

Inoculated animals, guinea-pigs and rabbits offer no difference in symptoms, so far as I can detect in a moderate number of trials, except that the hog cholera germ does not act quite so quickly. Pathological changes the same, undistinguishable except that the heart is more often in systole, and the trachea is suffused with small hemorrhages. The value of the last symptom may be quite considerable. Twenty-five guinea-pigs and rabbits killed with my cultures did not show it. Those killed with hog cholera all showed it. It must be noted, however, that the same is true of a guinea-pig killed by Schutz germ, which to my mind is identical with mine. I have not noticed œdema.

My sections of "Schwein seuche," from Austria, certainly give the impression of being something else. The bacteria are well stained by the Gram method, though the nuclei are well bleached, and appear as a "bipolar organism." It does not resemble any of my other material, and is characterized by taking the Gram method well. Löffler's germ is said to do the same, but none of my own cultures or that from Schutz's will do it in my hands, even though the nuclei are all left colored.

I am inclined to suspect a different germ but certainly cannot affirm it.

Hueppe's "Wild seuche" specimens offer a good many points of difference. They stained easily with anything, according to Dr. Jackson, who assisted Hueppe. As seen in sections, the germ occurs chiefly in clusters as a short bacillus, at times beautifully bipolar, but never looks like a micrococcus. Agar-agar culture preparations show small bacilli up to $3\eta$ long, uniformly stained except for a fine division line in many individuals; gelatin cultures show micrococci about $.5\eta$. In small animals the tendency to hemorrhage is evidently greater and the trachea affected. Unfortunately Hueppe has failed to give such detailed description as is to be desired, but has gone extensively into reasoning away discrepancies. Such methods are liable to lead to fallacies, as well shown in the hog cholera reviews in Baumgarten's Jähresberichte. Hueppe's chief argument, similarity of effect on small animals, is thrown out of court by the fact that two distinct plants—hog cholera and Loeffler's—cause the same symptoms.

Turning to a general consideration of the contagious diseases of hogs the world over, we meet a most difficult question, and all opinions must be regarded as tentative until all the various kinds (?) of germs have been collected and compared simultaneously by a competent person. As already stated, very few of the descriptions given are sufficient to rely upon. Ruling out swine erysipelas, rouget or Rothlauf, a disease caused by a small distinct bacillus not occurring in America, the positions taken by various authors at various times may be classed under four heads.

1. That there is but one hog plague—whatever its name, and this common to Europe and America.

2. That there are two diseases of hogs, properly represented by Loeffler's germ and the germ of hog cholera. Who first properly described this germ I shall not endeavor to say, though I believe Detmers was the first to call attention to it, long before the time of modern bacteriological methods.

3. That there are four hog plagues.—Reitsch.

4. That in Europe there is one disease common to all the smaller domestic animals which has generally been regarded as many diseases in as many animals. Thus, rabbit septicæmia, chicken cholera, schwein seuch (Loeffler), wild seuche, are all one and the same disease. (Hueppe.) The common American disease, hog cholera, not referred to.

The first of these positions is now, I believe, discarded by all. The fourth is to my mind probably wrong. At all events the numerous series of preparations of Hueppe's wild seuche at my disposal do not agree with my preparation of anything, chicken cholera, rabbit septicæmia, swine seuche (Loeffler), or my Austrian material.

There remains then the question, are there more than two diseases? This I cannot answer positively, but believe that there probably are.

As is now well known, there are a whole series of germs forming properly a genus all more or less "belted" or "bi-polar" in nature, causing disease in this or that animal, the diseases in all cases being very much alike, and where occurring in the same species of animal, offering few if any pathological points of distinction. As representative of this group may be mentioned the germs of hog cholera or swine plague, chicken cholera—rabbit septicæmia—schwein seuche—swine plague—corn disease, Texan cattle fever, wild seuche. These and perhaps others, I believe, are responsible for the utter confusion which reigns in the literature of the subject.

To sum up, the two epidemics studied by me were due to a small bi-polar germ identical with sample of swine plague received from Washington and culture of Loeffler's germ from Schutz, per Billings. No hog cholera or swine plague bacilli occurred. No characteristic lesions have been observed, the pathology pointing to a general infection, tending to chiefly affect the lungs and intestines.

Calves may contract the disease from swine. (See Gaultier as regards sheep.) There are undoubtedly several kinds of germs causing the same symptoms in such small experiment animals as are susceptible to them. But while only two germs of this class are known to infest hogs in the United States there may be others in Europe. (*e. g.*, Wild seuche.)

